# Association between early administration of mucoactive agents and in-hospital mortality in patients with pneumonia requiring mechanical ventilation: a nationwide cohort study

**DOI:** 10.1186/s40560-025-00826-7

**Published:** 2025-10-16

**Authors:** Akira Sasaki, Mikio Nakajima, Tomohiro Shinozaki, Yusuke Sasabuchi, Hiroyuki Ohbe, Richard H. Kaszynski, Yuya Kimura, Kojiro Morita, Tadahiro Goto, Yuki Aiyama, Izumi Nakayama, Hiroki Matsui, Kiyohide Fushimi, Hidenobu Ochiai, Hideo Yasunaga

**Affiliations:** 1 Department of Emergency and Critical Care Medicine, University of Miyazaki Hospital, Miyazaki, Japan; 2 Department of Emergency Medicine, Miyazaki Prefectural Miyazaki Hospital, Miyazaki, Japan; 3 Emergency and Critical Care Center, Tokyo Metropolitan Hiroo Hospital, 2-34-10, Ebisu, Shibuya-Ku, Tokyo 150-0013 Japan; 4 Department of Clinical Epidemiology and Health Economics, School of Public Health, The University of Tokyo, Tokyo, Japan; 5 Interfaculty Initiative in Information Studies, The University of Tokyo, Tokyo, Japan; 6 Department of Biostatistics, School of Public Health, Graduate School of Medicine, The University of Tokyo, Tokyo, Japan; 7 Department of Real-World Evidence, Graduate School of Medicine, The University of Tokyo, Tokyo, Japan; 8 Department of Emergency and Critical Care Medicine, Tohoku University Hospital, Sendai, Japan; 9 Department of Health Services Research, Graduate School of Medicine, The University of Tokyo, Tokyo, Japan; 10 Department of Nursing Administration and Advanced Clinical Nursing, Division of Health Sciences and Nursing, Graduate School of Medicine, The University of Tokyo, Tokyo, Japan; 11 Department of Health Data Science, Graduate School of Data Science, Yokohama City University, Kanagawa, Japan; 12 Department of Emergency and Critical Care Medicine, Faculty of Medicine, Shimane University, Shimane, Japan; 13 Department of Public Health, Yokohama City University School of Medicine, Yokohama, Japan; 14 Department of Health Policy and Informatics, Tokyo Medical and Dental University Graduate School of Medical and Dental Sciences, Tokyo, Japan

**Keywords:** Acute respiratory failure, Propensity score overlap weighting, Inverse probability of treatment weighting, Doubly robust, Mortality, Sputum

## Abstract

**Background:**

In patients with pneumonia requiring mechanical ventilation, increased airway secretions are associated with prolonged mechanical ventilation, but the effect of mucoactive agents remains unclear. The present study aimed to investigate the association between early administration of mucoactive agents and in-hospital mortality in patients with pneumonia requiring mechanical ventilation.

**Methods:**

We conducted a retrospective cohort study using the Japanese Diagnosis Procedure Combination database, a nationwide inpatient database. Adult patients were eligible if they had a primary diagnosis of pneumonia and required invasive mechanical ventilation within 2 days of admission, with ventilation continued for ≥ 2 days between April 2012 and March 2023. Patients were divided into those who received at least one mucoactive agent within 2 days after the initiation of mechanical ventilation (mucoactive agent group) and those who did not (control group). Mucoactive agents included ambroxol (oral), bromhexine (oral, intravenous and nebulized), fudosteine (oral), carbocisteine (oral) and N-acetylcysteine (nebulized). We performed a propensity score overlap weighting analysis to compare in-hospital mortality. The number of ventilator-free days at 28 days was assessed as a secondary outcome. We also performed sensitivity analyses using inverse probability of treatment weighting, generalized estimating equations, and doubly robust methods.

**Results:**

Eligible patients (*n* = 10,942) were categorized into the mucoactive agent group (*n* = 2246) or control group (*n* = 8696). The most commonly prescribed mucoactive agent was carbocisteine (oral). After overlap weighting, in-hospital mortality was significantly lower in the mucoactive agent group than in the control group (25.2% vs. 27.5%; risk difference, − 2.3%; 95% confidence interval, − 4.4% to − 0.3%; *p* = 0.028). Ventilator-free days at 28 days did not significantly differ between the mucoactive agent group and the control group. Sensitivity analyses yielded similar results.

**Conclusions:**

In patients with ventilated pneumonia, early administration of mucoactive agents was associated with lower in-hospital mortality.

**Graphical Abstract:**

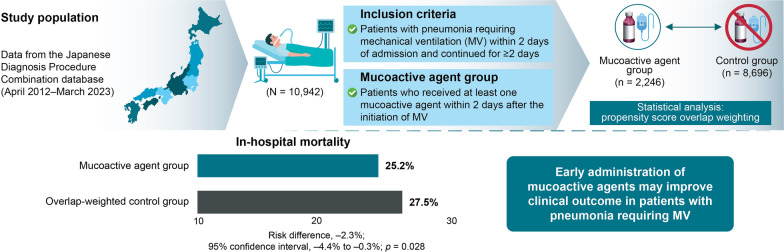

**Supplementary Information:**

The online version contains supplementary material available at 10.1186/s40560-025-00826-7.

## Background

Prolonged mechanical ventilation is associated with poor clinical outcomes [1–3], and therefore, the recent guidelines advocate for early liberation from mechanical ventilation [4]. Patients placed on mechanical ventilation often develop mucus accumulation due to impaired mucociliary transport [5, 6]. In patients who successfully pass a spontaneous breathing trial, successful extubation is associated with several factors that influence airway clearance, such as coughing and the amount of airway secretions [7, 8].

In a recent survey of critically ill patients in the United Kingdom, 41% of mechanically ventilated patients received at least one mucoactive agent [9]. A previous meta-analysis compared mucoactive agents versus placebo in patients with mechanical ventilation for acute respiratory failure and revealed that the use of mucoactive agents did not improve outcomes, including duration of mechanical ventilation, mortality or ventilator-free days at day 28 [10].

Various guidelines do not specify whether mucoactive agents should be administered to patients requiring mechanical ventilation [11–14]. Pneumonia is characterized by increased sputum production [15], and has been associated with the risk of prolonged mechanical ventilation [1, 3]. However, the efficacy of administering mucoactive agents in patients with pneumonia requiring mechanical ventilation has not been evaluated on a large-scale level. We hypothesized that early administration of mucoactive agents in patients with pneumonia requiring mechanical ventilation after hospitalization is associated with reduced in-hospital mortality.

## Methods

### Data source

We conducted a retrospective cohort study using the Japanese Diagnosis Procedure Combination (DPC) database. The database includes administrative claims data and discharge abstracts from more than 1786 acute-care hospitals and all 82 university hospitals [16]. The database includes the following information for each patient: age; sex; body mass index at admission; level of consciousness; main and subcategorized secondary diagnosis; pre-existing comorbidities at admission; post-admission complications coded with International Classification of Diseases, 10th Revision (ICD-10) codes and text data entered in Japanese. Medical procedures, including types of surgery (coded with original Japanese codes), daily records of drug administration and devices used, length of hospital stay, and discharge status, were also included [16].

### Patient selection

We identified patients with pneumonia requiring invasive mechanical ventilation as the primary diagnosis and with a hospital discharge date between April 2012 and March 2023. We included patients with a primary diagnosis of pneumonia and patients requiring invasive mechanical ventilation within 2 days of admission and continued for ≥ 2 days. Pneumonia was defined as ICD-10 codes, J09–18, J69, and U071 (Supplementary Table 1). The validity of pneumonia diagnoses recorded in the DPC database has been previously evaluated and shown to have high specificity for bacterial (94.8%) and aspiration pneumonia (99.2%) [17].

We excluded (i) patients aged < 18 years, (ii) those with hospital-acquired pneumonia, (iii) those with cardiac arrest upon hospital arrival or within 1 day of admission, (iv) those with readmission, (v) patients for whom mechanical ventilation was not initiated within 2 days of admission, (vi) those who were not placed on mechanical ventilation for more than 2 days, (vii) those who were discharged within 3 days of admission (to avoid immortal time bias), and (viii) those with missing data on the severity of pneumonia, which attending physicians are required to enter for all patients admitted for pneumonia. No other variables had missing values apart from this severity score. Severity of pneumonia at admission was assessed based on components derived from the A-DROP (age, dehydration, respiration, disorientation, and blood pressure) system [18]. This scoring system is similar to the CURB-65 system of the British Thoracic Society and useful in assessing the severity of community-acquired pneumonia [19].

### Exposure and outcome

Patients were divided into two groups: (i) patients who received at least one mucoactive agent within 2 days after the initiation of mechanical ventilation (mucoactive agent group) and (ii) patients who did not receive any mucoactive agent within 2 days after the initiation of mechanical ventilation (control group). We selected a 2-day window to define early administration of mucoactive agents. Although a 24-h timeframe might be more clinically intuitive, the DPC database captures treatment timing only on a calendar-day basis, not by hour. For instance, if mechanical ventilation is initiated in the afternoon of the admission day and mucoactive agents are administered the next morning, this would be recorded as the second calendar day. To account for this limitation while approximating early use, we adopted a 2-day definition. Mucoactive agents included ambroxol (oral), bromhexine (oral, intravenous and nebulized), fudosteine (oral), carbocisteine (oral) and N-acetylcysteine (nebulized) [20]. The primary outcome was in-hospital mortality. Secondary outcome was ventilator-free days at 28 days of admission. Ventilator-free days at 28 days were defined as 28 minus the number of days on mechanical ventilation for patients who were successfully liberated within this period. Patients who died within 28 days or remained on mechanical ventilation for the entire 28 days were assigned a value of 0 [21].

### Variables

Variables included age, sex, body mass index, transfer via ambulance, teaching hospital, intensive care unit (ICU) admission, Charlson comorbidity index, comorbidities at admission (hypertension, diabetes mellitus, chronic obstructive pulmonary disease, asthma, bronchiectasis, interstitial lung disease), consciousness level on admission, cause of pneumonia, parameters of A-DROP on admission, immunocompromised status, severity of pneumonia on admission and the need for certain procedures and medications within 2 days of admission (renal replacement therapy, extracorporeal membrane oxygenation, vasopressors, immunoglobulin, albumin, steroid, antithrombin, recombinant human soluble thrombomodulin, sivelestat sodium, transfusion, and intravenous antibiotics). To quantify the extent of comorbidities, the Charlson comorbidity index is calculated by converting each comorbidity defined by its corresponding ICD-10 code into a numerical score and subsequently calculating the total sum of these scores [22]. The Charlson comorbidity index was categorized into four groups: 0, 1, 2, and ≥ 3 [23]. The details of comorbidities at admission are shown in Supplementary Table 2. Consciousness level on admission was recorded via the Japan Coma Scale, which is the most frequently adopted method for evaluating level of consciousness in Japan. A previous study demonstrated that the Japan Coma Scale and Glasgow Coma Scale were well-correlated [24]. Details of the variables are listed in Supplementary Table 3.

### Statistical analysis

We used a propensity score overlap weighting method to adjust for the unbalanced backgrounds between the mucoactive agent and the control groups. Overlap weights assigned greater weight to subgroups with propensity scores around 0.5, where clinical equipoise is most likely. They also achieve exact mean balance for all covariates included in the propensity score model [25, 26]. A logistic model was used to estimate propensity scores for receiving mucoactive agents, using the patient background characteristics, medications and interventions performed within 2 days after the initiation of mechanical ventilation. We additionally adjusted for individual component variables (e.g., diabetes mellitus and chronic pulmonary disease) in parallel with composite indices (e.g., Charlson Comorbidity Index) to mitigate potential model misspecification arising from discrepancies between the regression coefficients and the prespecified relative weights embedded in these scores. Likewise, both the Japan Coma Scale and the A-DROP score, which share a consciousness assessment component, were included simultaneously for the same reason. This approach was intended to enhance the robustness of the covariate adjustment. Overlap weighting balances the covariates between treated and untreated groups by weighting each patient by 1 − propensity score (for treated patients) or propensity score (for untreated patients). This approach minimizes the influence of the data with extreme propensity scores (close to 0 or 1), which helps improve precision compared to inverse probability of treatment weighting (IPTW) targeting the overall population [25, 26]. We calculated the risk differences in in-hospital mortality and 95% robust confidence intervals (CIs).

We conducted three sensitivity analyses to confirm the results of the primary analysis. First, we performed an IPTW analysis by the stabilized weights for the overall population [27]. Second, the covariates were adjusted for in the multivariable linear model, which was estimated by generalized estimating equations to obtain the cluster–robust variance accounting for hospitals as clusters. Third, we estimated the multivariable linear model with the stabilized weights, in which a coefficient of the exposure (i.e., mucoactive agents) provides the “doubly robust” estimate of risk difference when product terms are omitted [28].

Continuous variables were reported as mean and standard deviation and categorical variables were reported as percentage. All CIs and *p* values were obtained through cluster–robust (for the multivariable-adjusted model) or robust variance estimator (for other methods). Two-sided *p* values of < 0.05 were considered significant. All analyses were performed using Stata MP18 (StataCorp, College Station, TX).

## Results

**Fig. 1:**
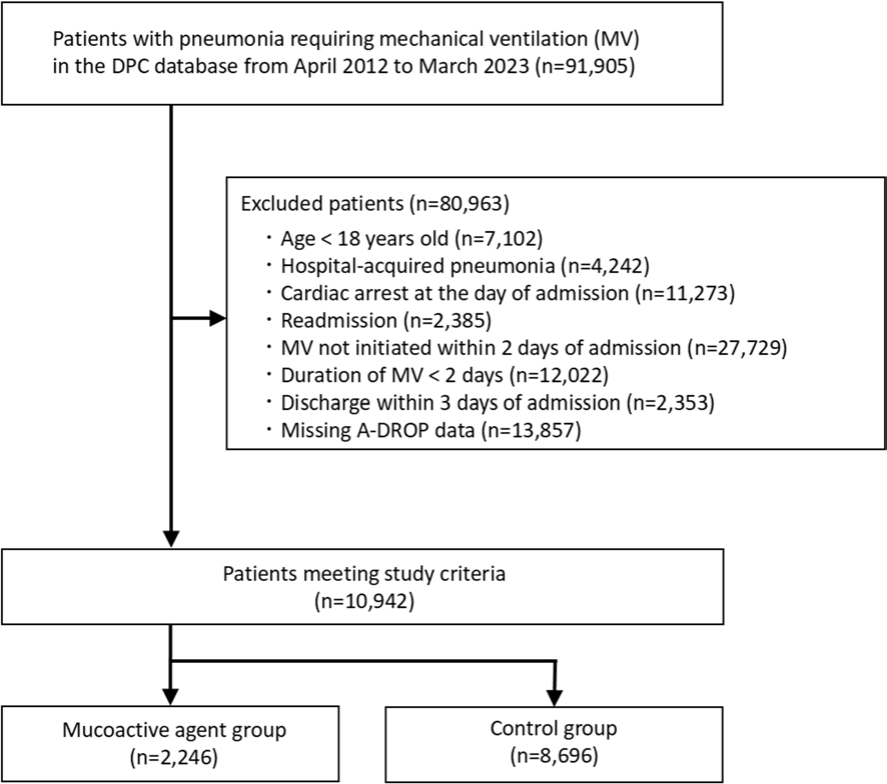
Flow of patient selection

The flowchart for patient selection is presented in Fig. 1. We identified 10,942 eligible patients during the study period, including 2246 patients in the mucoactive agent group and 8696 patients in the control group. The breakdown of mucoactive agents prescribed in the mucoactive agent group is shown in Supplementary Table 4. The most commonly prescribed mucoactive agent was carbocisteine (oral), followed by bromhexine (nebulized). Overall, 26.7% of patients in the mucoactive agent group received more than one type of mucoactive agent (Supplementary Table 5).

Table 1 demonstrates the summary of baseline characteristics of patients before and after overlap weighting (all variables were listed in Supplementary Table 3). The C-statistic was 0.66 in the propensity score models. In the cohort before overlap weighting, the mucoactive agent group was younger and included more patients with chronic obstructive pulmonary disease and asthma. The control group tended to have greater illness severity, characterized by a higher proportion of ICU admissions, worse level of consciousness at admission, more frequent dehydration, poorer oxygenation status, and more frequent use of central venous catheters and vasopressors. After adjusting for measured confounders using overlap weights, characteristics were well-balanced between the groups.

**Table 1:** Baseline patient characteristics before and after propensity score overlap weighting

	Before propensity score overlap weighting			After propensity score overlap weighting		
Variables	Mucoactive agent group (n = 2246)	Control group (n = 8696)	ASD	Mucoactive agent group	Control group	ASD
Age, years, mean	73	76	19%	74	74	0%
Sex, male	1478 (65.8%)	5661 (65.1%)	2%	65%	65%	0%
Intensive care unit	955 (42.5%)	4180 (48.1%)	11%	44%	44%	0%
Comorbidities						
Charlson comorbidity index						
0	507 (22.6%)	2132 (24.5%)	5%	23%	23%	0%
1	776 (34.6%)	2999 (34.5%)	0%	34%	34%	0%
2	530 (23.6%)	1924 (22.1%)	4%	23%	23%	0%
≥ 3	433 (19.3%)	1641 (18.9%)	1%	19%	19%	0%
Hypertension	624 (27.8%)	2447 (28.1%)	1%	28%	28%	0%
Diabetes mellitus	412 (18.3%)	1864 (21.4%)	8%	19%	19%	0%
COPD	728 (32.4%)	1652 (19.0%)	31%	29%	29%	0%
Asthma	364 (16.2%)	697 (8.0%)	25%	14%	14%	0%
Bronchiectasis	82 (3.7%)	148 (1.7%)	12%	3%	3%	0%
Interstitial lung disease	99 (4.4%)	480 (5.5%)	5%	5%	5%	0%
Japan Coma Scale						
0 (alert)	1344 (59.8%)	4423 (50.9%)	18%	58%	58%	0%
One digit (dizziness)	497 (22.1%)	2087 (24.0%)	4%	23%	23%	0%
Two digits (somnolence)	181 (8.1%)	909 (10.5%)	8%	9%	9%	0%
Three digits (Coma)	224 (10.0%)	1277 (14.7%)	14%	11%	11%	0%
Cause of pneumonia						
Viral pneumonia	7 (0.3%)	9 (0.1%)	5%	0%	0%	0%
Influenza virus	34 (1.5%)	164 (1.9%)	3%	2%	2%	0%
COVID-19	8 (0.4%)	72 (0.8%)	6%	0%	0%	0%
Bacterial pneumonia	930 (41.4%)	3370 (38.8%)	5%	41%	41%	0%
Streptococcus pneumoniae	150 (6.7%)	653 (7.5%)	3%	7%	7%	0%
Haemophilus influenzae	49 (2.2%)	116 (1.3%)	7%	2%	2%	0%
Chlamydophila pneumoniae	5 (0.2%)	10 (0.1%)	3%	0%	0%	0%
Aspiration pneumonia	162 (7.2%)	634 (7.3%)	0%	7%	7%	0%
Others	1007 (44.8%)	4018 (46.2%)	3%	45%	45%	0%
A-DROP components						
Blood urea nitrogen ≥ 21 mg/dL or dehydration	1268 (56.5%)	5498 (63.2%)	14%	59%	59%	0%
SpO_2_ > 90% (room air)	421 (18.7%)	1448 (16.7%)	6%	18%	18%	0%
SpO_2_ ≤ 90% (room air) or needed F_I_O_2_ ≤ 35% to maintain SpO_2_ > 90%	730 (32.5%)	2445 (28.1%)	10%	31%	31%	0%
SpO_2_ ≤ 90% (room air) or needed F_I_O_2_ > 35% to maintain SpO_2_ > 90%	1095 (48.8%)	4803 (55.2%)	13%	50%	50%	0%
Orientation disturbance	798 (35.5%)	3857 (44.4%)	18%	38%	38%	0%
Systolic blood pressure ≤ 90 mmHg	344 (15.3%)	1609 (18.5%)	9%	16%	16%	0%
Immunocompromised status	320 (14.2%)	1374 (15.8%)	4%	15%	15%	0%
Healthcare-associated pneumonia	511 (22.8%)	1817 (20.9%)	5%	22%	22%	0%
Interventions within 2 days of admission						
Central venous catheter	372 (16.6%)	1797 (20.7%)	11%	18%	18%	0%
Intra-aortic balloon pump	0 (0.0%)	2 (0.0%)	2%	0%	0%	0%
High flow nasal cannula	12 (0.5%)	69 (0.8%)	3%	1%	1%	0%
NIV	4 (0.2%)	2 (0.0%)	5%	0%	0%	0%
Renal replacement therapy	30 (1.3%)	202 (2.3%)	7%	1%	1%	0%
PMX–DHP	2 (0.1%)	12 (0.1%)	2%	0%	0%	0%
Medication within 2 days of admission						
Corticosteroid	571 (25.4%)	1834 (21.1%)	10%	24%	24%	0%
Steroid pulse therapy	341 (15.2%)	1091 (12.5%)	8%	14%	14%	0%
Vasopressor	393 (17.5%)	1933 (22.2%)	12%	19%	19%	0%
Neuromuscular blocking agents	178 (7.9%)	971 (11.2%)	11%	9%	9%	0%

*ASD* absolute standardized difference, *COPD* chronic obstructive pulmonary disease, *COVID-19* coronavirus disease 2019, *A-DROP* severity score consisting of age, dehydration, respiration, orientation, and blood pressure, *CRP* C-reactive protein, *NIV* non-invasive ventilation, *PMX–DHP* polymyxin B-immobilized fiber column direct hemoperfusion

Table 2 shows in-hospital mortality and ventilator-free days at 28 days of admission. After overlap weighting, in-hospital mortality was significantly lower in the mucoactive agent group than in the control group (mucoactive agent group 25.2% vs. control group 27.5%; risk difference, − 2.3%; 95% CI, − 4.4% to − 0.3%; *p* = 0.028). Ventilator-free days at 28 days did not significantly differ between the mucoactive agent group and the control group (12.4 days vs. 12.6 days; difference, − 0.21 day; 95% CI, − 0.71 to 0.29 day; *p* = 0.414).

**Table 2:** Differences in outcomes between mucoactive agent and control groups with different adjustment methods

	Mucoactive agent group	Control group	Difference	(95% CI)*	*p* value*
In-hospital mortality, %					
Crude risk	24.1	30.2	–6.1	(–8.1 to –4.1)	< 0.001
Adjusted risk†					
Overlap weighting	25.2	27.5	–2.3	(–4.4 to –0.3)	0.028
IPTW	27.5	29.4	–2.2	(–4.5 to 0.1)	0.062
Multivariable-adjusted	–	–	–2.3	(–4.3 to –0.3)	0.025
IPTW multivariable-adjusted (doubly robust)	–	–	–2.1	(–4.3 to –0.0)	0.050
Ventilator-free days, days (SD)					
Mean	12.5 (10.4)	12.4 (10.7)	0.15	(–0.24 to 0.63)	0.553
Adjusted mean†					
Overlap weighting	12.4 (10.5)	12.6 (10.6)	–0.21	(–0.71 to 0.29)	0.414
IPTW	12.2 (10.5)	12.5 (10.7)	–0.27	(–0.81 to 0.26)	0.318
Multivariable-adjusted	–	–	–0.24	(–0.74 to 0.25)	0.339
IPTW multivariable-adjusted (doubly robust)	–	–	–0.28	(–0.79 to 0.23)	0.290

*CI* confidence interval, *GEE* generalized estimating equation, *IPTW* inverse probability of treatment weighting, *SD* standard deviation

^*^All CIs and *p* values were obtained through cluster–robust (multivariable-adjusted) or robust variance estimator (other methods). The cluster–robust variance was estimated through generalized estimating equations for linear models accounting for hospitals as clusters

^†^Adjusted variables in the outcome models (for GEE and doubly robust) and the propensity score logistic models (for IPTW, overlap weighting, and doubly robust) are described in the main text

The results of the sensitivity analyses are shown in Table 2. In the stabilized IPTW analyses, there were no significant differences in in-hospital mortality (risk difference, − 2.2%; 95% CI, − 4.5% to 0.1%; *p* = 0.062). In the multivariable-adjusted analysis, the mucoactive agent group was significantly associated with lower in-hospital mortality (risk difference, − 2.3%; 95% CI, − 4.3% to − 0.3%; *p* = 0.025). In the doubly robust, IPTW multivariable-adjusted analysis, the mucoactive agent group was significantly associated with lower in-hospital mortality (risk difference, − 2.1%; 95% CI, − 4.3% to − 0.0%; *p* = 0.050). Ventilator-free days did not significantly differ between the groups in all sensitivity analyses.

## Discussion

In this nationwide study, we examined the association between early administration of mucoactive agents and the outcomes in patients with pneumonia requiring mechanical ventilation, using propensity score overlap weighting. Early administration of mucoactive agents was significantly associated with lower in-hospital mortality. This reduction in mortality appears clinically meaningful in this high-mortality population. The risk difference of –2.3% corresponds to a number needed to treat of approximately 43, meaning that treating 43 patients with a mucoactive agent—an inexpensive and generally well-tolerated medication—would be expected to prevent one death.

Our findings were in contrast to those in a recent meta-analysis which showed the use of mucoactive agents in critically ill patients receiving mechanical ventilation for acute respiratory failure did not improve mortality, ventilator-free days at day 28, duration of ventilation and duration of hospital stay, although use of mucoactive agents reduced the duration of ICU stay (− 3.2 days) [10]. The meta-analysis included 13 randomized controlled trials (1712 patients) and investigated four mucoactive agents, including nebulized N-acetylcysteine (8 trials), heparin (3 trials), hypertonic saline (1 trial) and intravenous ambroxol (1 trial).

Considering the differences in the method of delivery and the type of mucoactive agents reported in previous studies, the mode of administration for mucoactive agents may have contributed to the improved clinical outcomes in this study. Though the above-mentioned meta-analysis investigated mucoactive agents (N-acetylcysteine, heparin, hypertonic saline and ambroxol) which were administered either via nebulizer or intravenously, this study included a number of mucoactive agents that were administered orally, such as ambroxol, bromhexine, fudosteine, and carbocisteine. With regard to bromhexine, it should be noted that the drug can also be administered via nebulizer or intravenously. Smaller studies included in the meta-analysis reported that hypertonic saline nebulization or intravenous ambroxol shortened the duration of mechanical ventilation [10]. Because hypertonic saline nebulization is not commonly used in patients under mechanical ventilation and ambroxol cannot be administered intravenously in Japan, we did not evaluate the effectiveness of hypertonic saline nebulization and intravenous ambroxol [20]. Aerosol delivery during mechanical ventilation is often compromised by circuit flows, positive pressure, and heating and humidification, all of which markedly reduce deposition efficiency [29]. By contrast, oral administration provides more consistent drug delivery under mechanical ventilation and is generally better tolerated. Notably, carbocisteine was not evaluated in the prior meta-analysis, yet it was the most frequently used mucoactive agent in our cohort and may, therefore, have contributed to the observed benefit. Beyond its mucoregulatory effects, carbocisteine also possesses antioxidant and anti-inflammatory properties [30], which may partly explain the improved clinical outcomes observed in the mucoactive agent group, although these mechanisms were not directly assessed in this study.

To the best of our knowledge, this is the first large-scale study to investigate the effectiveness of mucoactive agents in pneumonia patients with mechanical ventilation. We included only patients with pneumonia who had a high risk for airway secretion retention. In the above-mentioned meta-analysis, 9 trials included patients with acute respiratory distress syndrome and 4 trials with a mixed population with acute respiratory failure, including those with sepsis, pneumonia, trauma, out-of-hospital cardiac arrest, heart failure and aspiration. Patients with pneumonia only accounted for approximately 14% of all patients in the meta-analysis [10]. Therefore, mucoactive agents may be effective in a specific subset of patients such as those with pneumonia. This study was not designed to determine which subgroups benefit most from mucoactive agents, and further research is needed to clarify this point.

In the present study, early administration of mucoactive agents was associated with lower in-hospital mortality, but no corresponding improvement was observed in ventilator-free days. This discrepancy may reflect differences in the clinical course of survivors. A greater proportion of patients in the mucoactive agent group survived but were discharged while still receiving mechanical ventilation within 28 days (9.7% vs. 8.0%; *p* = 0.013) and, therefore, had ventilator-free days of zero despite survival (12.1% vs. 10.1%; *p* = 0.009), compared with the control group. These findings suggest that many patients survived but required prolonged mechanical ventilation, potentially offsetting any observable gain in ventilator-free days. Mucoactive agents may have improved survival by preventing fatal complications such as airway obstruction caused by retained secretions in the endotracheal tube or trachea, reducing partial airway obstruction that can impair gas exchange, and lowering the risk of secondary infections, including ventilator-associated pneumonia. This mechanism could reduce mortality without necessarily shortening the duration of mechanical ventilation.

We performed propensity score overlap weighting analysis as the primary analysis and IPTW, generalized estimating equation and doubly robust analyses for the sensitivity analyses. In the propensity score overlap weighting, generalized estimating equation and doubly robust analyses, administration of mucoactive agents was significantly associated with lower in-hospital mortality, but not in the IPTW analysis. Although there was no significant difference in in-hospital mortality in the IPTW, the point estimate of adjusted risk difference (approximately − 2%) was similar to other analyses and considered clinically significant. This discrepancy is likely due to inherent limitations of IPTW, particularly its susceptibility to extreme propensity scores that generate highly variable weights. Such unstable weights inflate the variance and widen confidence intervals, potentially obscuring clinically meaningful differences [31, 32]. By contrast, overlap weighting mitigates the influence of extreme propensity scores, achieves better covariate balance, and yields more stable effect estimates with smaller variance. Although overlap weighting emphasizes the subpopulation with greater clinical equipoise (i.e., those with estimated propensity scores closer to 0.5), thereby shifting the target population from the overall cohort [32], we selected it as the primary analysis to take advantage of its improved statistical precision.

We defined the mucoactive agent group as patients who received mucoactive agents within 2 days after the initiation of mechanical ventilation. This definition would imply that we included patients who received mucoactive agents as routine practice after the initiation of mechanical ventilation. A recent study revealed a high prevalence of mucoactive agent use in mechanically ventilated patients, with the most common indication for viscous airway secretions. The clinical aim for utilizing mucoactive agents was to reduce the duration of mechanical ventilation [9]. Further studies to evaluate early engagement with mucoactive agents are needed for pneumonia patients with mechanical ventilation and viscous airway secretions.

The present study has several limitations. First, this is a retrospective study using routinely collected data in Japan. Because the diagnoses were identified using ICD-10 codes, some degree of misclassification is possible. Nevertheless, a prior validation study of the DPC database demonstrated high specificity for pneumonia diagnoses, suggesting that any misclassification bias is likely minimal [17]. To date, no published validation studies have systematically evaluated the diagnostic accuracy of viral pneumonias–most notably coronavirus disease 2019 (COVID-19)–despite their clinical and public health importance. Second, the DPC database lacks information on the quantity and composition of the sputum encountered for each patient. Therefore, we cannot definitively state that the mucoactive agents were administered to patients presenting with sputum or viscous airway secretions. However, it is difficult to objectively assess sputum in critically ill patients [8]. Third, the database does not include detailed clinical information, such as symptoms, vital signs, laboratory data, culture, image findings and oxygenation, as well as factors, such as physician preferences and hospital-specific protocols, which may have inadvertently introduced bias. However, because we focused on patients with pneumonia requiring mechanical ventilation and adjusted for severity by A-DROP, we consider that the severity of pneumonia was comparable between the two groups. Fourth, although some patients underwent intra-aortic balloon pump support or polymyxin B-immobilized fiber column direct hemoperfusion—interventions that may reflect different pathophysiological conditions, such as cardiogenic shock or severe abdominal septic shock—their influence on the overall study findings was likely minimal given the small number of such cases. Likewise, the number of COVID-19 patients in our cohort was very small, likely reflecting the fact that cases with COVID-19 (DPC disease classification code, 180030) are reimbursed on a fee-for-service basis rather than the DPC-based payment system in Japan. In such cases, the recording of A-DROP components is not mandatory, and these cases were excluded from this study because of missing A-DROP data. COVID-19 is often characterized by distinct pathological features and a clinical course different from typical pneumonia; thus its impact on our results was also likely negligible. Moreover, because fiscal year was included as a variable in the propensity score model, temporal changes in clinical practice during the pandemic (e.g., ventilator strategies, prone positioning, and adjunctive therapies) were expected to be balanced between the groups. Fifth, this study lacks data on humidification methods used in the ventilator circuits; however, a previous study suggested that humidification methods have limited impacts on mortality and duration of mechanical ventilation [20]. Sixth, we had no information pertaining to adverse events associated with the administration of mucoactive agents. The reported side effects of mucoactive agents include gastrointestinal disorders, dizziness, headache, and erythema, and they would not adversely affect the clinical outcome [33]. Finally, our cohort included nearly equal proportions of ICU and non-ICU patients, reflecting real-world practice in Japan [34]. While this differs from settings, where mechanical ventilation is performed almost exclusively in ICUs, the inclusion of both ICU and non-ICU patients may limit external validity of our findings when extrapolated to other healthcare systems.

## Conclusion

This nationwide retrospective cohort study demonstrated that early administration of mucoactive agents may improve clinical outcome in patients with pneumonia requiring mechanical ventilation.

## Supplementary Information

Additional file 1.

## Data Availability

Not applicable.

## Abbreviations

DPC: Diagnosis procedure combination
ICD-10: International Classification of Diseases, Tenth Revision
ICU: Intensive care unit
IPTW: Inverse probability of treatment weighting
CI: Confidence interval
COVID-19: Coronavirus disease 2019
