# Supplementary material for: Association between early administration of mucoactive agents and in-hospital mortality in patients with pneumonia requiring mechanical ventilation: a nationwide cohort study
Source: J Intensive Care. 2025 Oct 16;13:57. doi: 10.1186/s40560-025-00826-7 (PMC12532859; doi:10.1186/s40560-025-00826-7)
Supplement: Supplementary file 1 — Additional file 1. [file 40560_2025_826_MOESM1_ESM.docx]

**Table S1**. ICD-10 codes for pneumonia

| ICD-10 code | Disease |
| --- | --- |
| J09 | Influenza due to identified zoonotic or pandemic influenza virus |
| J10 | Influenza due to identified seasonal influenza virus |
| J11 | Influenza, virus not identified |
| J12 | Viral pneumonia, not elsewhere classified |
| J13 | Pneumonia due to Streptococcus pneumoniae |
| J14 | Pneumonia due to Haemophilus influenzae |
| J15 | Bacterial pneumonia, not elsewhere classified |
| J16 | Pneumonia due to other infectious organisms, not elsewhere classified |
| J17 | Pneumonia in diseases classified elsewhere |
| J18 | Pneumonia, organism unspecified |
| J69 | Pneumonitis due to food and vomit |
| U071 | COVID-19, virus identified |

Abbreviations: COVID-19, coronavirus disease 2019

**Table S2**. ICD-10 codes for comorbidities

| Variables | ICD-10 |
| --- | --- |
| Hypertension | I10–13, I15 |
| Diabetes mellitus | E10–14 |
| COPD | J41–J44 |
| Asthma | J45, J46 |
| Bronchiectasis | J47 |
| Interstitial lung disease | J84 |

Abbreviations: COPD, chronic obstructive pulmonary disease

**Table S3**. Detailed baseline patient characteristics before and after propensity score overlap weighting

|  | Before propensity score overlap weighting | | |  | After propensity score overlap weighting | | |
| --- | --- | --- | --- | --- | --- | --- | --- |
| Variables | Mucoactive agent group (n=2,246) | Control group (n=8,696) | ASD |  | Mucoactive agent group | Control group | ASD |
| Age, years, mean | 73 | 76 | 19% |  | 74 | 74 | 0% |
| Sex, male | 1478 (65.8%) | 5661 (65.1%) | 2% |  | 65% | 65% | 0% |
| Body mass index |  |  |  |  |  |  |  |
| < 18.5 kg/m² | 700 (31.2%) | 2430 (27.9%) | 7% |  | 30% | 30% | 0% |
| 18.5–24.9 kg/m² | 969 (43.1%) | 3841 (44.2%) | 2% |  | 43% | 43% | 0% |
| 25-29.9 kg/m² | 226 (10.1%) | 980 (11.3%) | 4% |  | 11% | 11% | 0% |
| ≥ 30 kg/m² | 107 (4.8%) | 430 (4.9%) | 1% |  | 5% | 5% | 0% |
| Missing | 244 (10.9%) | 1015 (11.7%) | 3% |  | 11% | 11% | 0% |
| Ambulance use | 1373 (61.1%) | 6088 (70.0%) | 19% |  | 64% | 64% | 0% |
| Teaching hospital | 1865 (83.0%) | 7480 (86.0%) | 8% |  | 84% | 84% | 0% |
| Intensive care unit | 955 (42.5%) | 4180 (48.1%) | 11% |  | 44% | 44% | 0% |
| Comorbidities |  |  |  |  |  |  |  |
| Charlson comorbidity index |  |  |  |  |  |  |  |
| 0 | 507 (22.6%) | 2132 (24.5%) | 5% |  | 23% | 23% | 0% |
| 1 | 776 (34.6%) | 2999 (34.5%) | 0% |  | 34% | 34% | 0% |
| 2 | 530 (23.6%) | 1924 (22.1%) | 4% |  | 23% | 23% | 0% |
| ≥3 | 433 (19.3%) | 1641 (18.9%) | 1% |  | 19% | 19% | 0% |
| Hypertension | 624 (27.8%) | 2447 (28.1%) | 1% |  | 28% | 28% | 0% |
| Diabetes mellitus | 412 (18.3%) | 1864 (21.4%) | 8% |  | 19% | 19% | 0% |
| COPD | 728 (32.4%) | 1652 (19.0%) | 31% |  | 29% | 29% | 0% |
| Asthma | 364 (16.2%) | 697 (8.0%) | 25% |  | 14% | 14% | 0% |
| Bronchiectasis | 82 (3.7%) | 148 (1.7%) | 12% |  | 3% | 3% | 0% |
| Interstitial lung disease | 99 (4.4%) | 480 (5.5%) | 5% |  | 5% | 5% | 0% |
| Japan Coma Scale |  |  |  |  |  |  |  |
| 0 (alert) | 1344 (59.8%) | 4423 (50.9%) | 18% |  | 58% | 58% | 0% |
| 1-digits (dizziness) | 497 (22.1%) | 2087 (24.0%) | 4% |  | 23% | 23% | 0% |
| 2-digits (somnolence) | 181 (8.1%) | 909 (10.5%) | 8% |  | 9% | 9% | 0% |
| 3-digits (Coma) | 224 (10.0%) | 1277 (14.7%) | 14% |  | 11% | 11% | 0% |
| Cause of pneumonia |  |  |  |  |  |  |  |
| Viral pneumonia | 7 (0.3%) | 9 (0.1%) | 5% |  | 0% | 0% | 0% |
| Influenza virus | 34 (1.5%) | 164 (1.9%) | 3% |  | 2% | 2% | 0% |
| COVID-19 | 8 (0.4%) | 72 (0.8%) | 6% |  | 0% | 0% | 0% |
| Bacterial pneumonia | 930 (41.4%) | 3370 (38.8%) | 5% |  | 41% | 41% | 0% |
| Streptococcus pneumoniae | 150 (6.7%) | 653 (7.5%) | 3% |  | 7% | 7% | 0% |
| Haemophilus influenzae | 49 (2.2%) | 116 (1.3%) | 7% |  | 2% | 2% | 0% |
| Chlamydophila pneumoniae | 5 (0.2%) | 10 (0.1%) | 3% |  | 0% | 0% | 0% |
| Aspiration pneumonia | 162 (7.2%) | 634 (7.3%) | 0% |  | 7% | 7% | 0% |
| Others | 1007 (44.8%) | 4018 (46.2%) | 3% |  | 45% | 45% | 0% |
| A-DROP components |  |  |  |  |  |  |  |
| Blood urea nitrogen (BUN) ≥21 mg/dL or dehydration | 1268 (56.5%) | 5498 (63.2%) | 14% |  | 59% | 59% | 0% |
| SpO_2_ >90% (room air) | 421 (18.7%) | 1448 (16.7%) | 6% |  | 18% | 18% | 0% |
| SpO_2_ ≤90% (room air) or needed F_I_O_2_ ≤35% to  maintain SpO_2_ >90% | 730 (32.5%) | 2445 (28.1%) | 10% |  | 31% | 31% | 0% |
| SpO_2_ ≤90% (room air) or needed F_I_O_2_ >35% to  maintain SpO_2_ >90% | 1095 (48.8%) | 4803 (55.2%) | 13% |  | 50% | 50% | 0% |
| Orientation disturbance | 798 (35.5%) | 3857 (44.4%) | 18% |  | 38% | 38% | 0% |
| Systolic blood pressure ≤90 mmHg | 344 (15.3%) | 1609 (18.5%) | 9% |  | 16% | 16% | 0% |
| Immunocompromised status | 320 (14.2%) | 1374 (15.8%) | 4% |  | 15% | 15% | 0% |
| Pneumonia severity–defining factors | 804 (35.8%) | 3527 (40.6%) | 10% |  | 37% | 37% | 0% |
| Healthcare-associated pneumonia |  |  |  |  |  |  |  |
| No | 950 (42.3%) | 3656 (42.0%) | 1% |  | 42% | 42% | 0% |
| Yes | 511 (22.8%) | 1817 (20.9%) | 5% |  | 22% | 22% | 0% |
| Missing | 785 (35.0%) | 3223 (37.1%) | 4% |  | 36% | 36% | 0% |
| Interventions within 2 days of admission |  |  |  |  |  |  |  |
| Intra-arterial blood pressure monitoring | 526 (23.4%) | 2332 (26.8%) | 8% |  | 24% | 24% | 0% |
| Central venous catheter | 372 (16.6%) | 1797 (20.7%) | 11% |  | 18% | 18% | 0% |
| Intra-aortic balloon pump | 0 (0.0%) | 2 (0.0%) | 2% |  | 0% | 0% | 0% |
| Tube thoracostomy | 25 (1.1%) | 93 (1.1%) | 0% |  | 1% | 1% | 0% |
| High flow nasal cannula | 12 (0.5%) | 69 (0.8%) | 3% |  | 1% | 1% | 0% |
| NIV | 4 (0.2%) | 2 (0.0%) | 5% |  | 0% | 0% | 0% |
| Renal replacement therapy | 30 (1.3%) | 202 (2.3%) | 7% |  | 1% | 1% | 0% |
| PMX-DHP | 2 (0.1%) | 12 (0.1%) | 2% |  | 0% | 0% | 0% |
| Extracorporeal membrane oxygenation | 6 (0.3%) | 16 (0.2%) | 2% |  | 0% | 0% | 0% |
| Tube feeding | 104 (4.6%) | 259 (3.0%) | 9% |  | 4% | 4% | 0% |
| Medication within 2 days of admission |  |  |  |  |  |  |  |
| Corticosteroid | 571 (25.4%) | 1834 (21.1%) | 10% |  | 24% | 24% | 0% |
| Steroid pulse therapy | 341 (15.2%) | 1091 (12.5%) | 8% |  | 14% | 14% | 0% |
| Vasopressor | 393 (17.5%) | 1933 (22.2%) | 12% |  | 19% | 19% | 0% |
| Immunoglobulin | 26 (1.2%) | 83 (1.0%) | 2% |  | 1% | 1% | 0% |
| Albumin | 53 (2.4%) | 212 (2.4%) | 1% |  | 2% | 2% | 0% |
| Antithrombin | 6 (0.3%) | 33 (0.4%) | 2% |  | 0% | 0% | 0% |
| Sivelestat sodium | 60 (2.7%) | 181 (2.1%) | 4% |  | 3% | 3% | 0% |
| Recombinant human soluble thrombomodulin | 9 (0.4%) | 64 (0.7%) | 5% |  | 0% | 0% | 0% |
| Dexmedetomidine | 237 (10.6%) | 902 (10.4%) | 1% |  | 11% | 11% | 0% |
| Propofol | 251 (11.2%) | 1284 (14.8%) | 11% |  | 12% | 12% | 0% |
| Midazolam | 363 (16.2%) | 1609 (18.5%) | 6% |  | 17% | 17% | 0% |
| Fentanyl | 282 (12.6%) | 1458 (16.8%) | 12% |  | 13% | 13% | 0% |
| Neuromuscular blocking agents | 178 (7.9%) | 971 (11.2%) | 11% |  | 9% | 9% | 0% |
| Transfusion within 2 days of admission |  |  |  |  |  |  |  |
| Red cell transfusion | 33 (1.5%) | 198 (2.3%) | 6% |  | 2% | 2% | 0% |
| Fresh frozen plasma transfusion | 10 (0.4%) | 46 (0.5%) | 1% |  | 0% | 0% | 0% |
| Platelets transfusion | 4 (0.2%) | 22 (0.3%) | 2% |  | 0% | 0% | 0% |
| Antibiotic use within 2 days of admission |  |  |  |  |  |  |  |
| Penicillin | 1 (0.0%) | 9 (0.1%) | 2% |  | 0% | 0% | 0% |
| Ampicillin | 8 (0.4%) | 21 (0.2%) | 2% |  | 0% | 0% | 0% |
| Ampicillin/sulbactam | 552 (24.6%) | 1985 (22.8%) | 4% |  | 24% | 24% | 0% |
| Piperacillin | 27 (1.2%) | 69 (0.8%) | 4% |  | 1% | 1% | 0% |
| Piperacillin/Tazobactam | 499 (22.2%) | 1810 (20.8%) | 3% |  | 22% | 22% | 0% |
| Third-generation cephalosporin | 505 (22.5%) | 1848 (21.3%) | 3% |  | 22% | 22% | 0% |
| Ceftazidime | 51 (2.3%) | 110 (1.3%) | 8% |  | 2% | 2% | 0% |
| Fourth-generation cephalosporin | 49 (2.2%) | 145 (1.7%) | 4% |  | 2% | 2% | 0% |
| Carbapenem | 342 (15.2%) | 1586 (18.2%) | 8% |  | 16% | 16% | 0% |
| Fluoroquinolone | 154 (6.9%) | 720 (8.3%) | 5% |  | 7% | 7% | 0% |
| Glycopeptide | 43 (1.9%) | 205 (2.4%) | 3% |  | 2% | 2% | 0% |
| Linezolid | 4 (0.2%) | 22 (0.3%) | 2% |  | 0% | 0% | 0% |
| Tetracycline | 55 (2.4%) | 199 (2.3%) | 1% |  | 2% | 2% | 0% |
| Macrolide | 133 (5.9%) | 575 (6.6%) | 3% |  | 6% | 6% | 0% |
| Peramivir | 57 (2.5%) | 278 (3.2%) | 4% |  | 3% | 3% | 0% |
| Remdesivir | 2 (0.1%) | 5 (0.1%) | 1% |  | 0% | 0% | 0% |
| Antifungal drug | 21 (0.9%) | 83 (1.0%) | 0% |  | 1% | 1% | 0% |
| Fiscal year |  |  |  |  |  |  |  |
| 2012 | 191 (8.5%) | 681 (7.8%) | 3% |  | 9% | 9% | 0% |
| 2013 | 172 (7.7%) | 739 (8.5%) | 3% |  | 8% | 8% | 0% |
| 2014 | 201 (8.9%) | 839 (9.6%) | 2% |  | 9% | 9% | 0% |
| 2015 | 221 (9.8%) | 964 (11.1%) | 4% |  | 10% | 10% | 0% |
| 2016 | 250 (11.1%) | 1045 (12.0%) | 3% |  | 12% | 12% | 0% |
| 2017 | 269 (12.0%) | 985 (11.3%) | 2% |  | 12% | 12% | 0% |
| 2018 | 289 (12.9%) | 925 (10.6%) | 7% |  | 12% | 12% | 0% |
| 2019 | 264 (11.8%) | 876 (10.1%) | 5% |  | 11% | 11% | 0% |
| 2020 | 151 (6.7%) | 641 (7.4%) | 3% |  | 7% | 7% | 0% |
| 2021 | 139 (6.2%) | 555 (6.4%) | 1% |  | 6% | 6% | 0% |
| 2022 | 99 (4.4%) | 446 (5.1%) | 3% |  | 5% | 5% | 0% |

Abbreviations: ASD, absolute standardized difference; COPD, chronic obstructive pulmonary disease; COVID-19, coronavirus disease 2019; A-DROP, severity score consisting of age, dehydration, respiration, orientation, and blood pressure; CRP, C-reactive protein; NIV, non-invasive ventilation; PMX-DHP, polymyxin B immobilized fiber column direct hemoperfusion

**Table S4**. Frequency of mucoactive agents used

|  | |
| --- | --- |
| Mucoactive agents | n (%) |
| Carbocisteine (oral) | 871 (38.8) |
| Bromhexine (nebulized) | 747 (33.3) |
| Ambroxol (oral) | 633 (28.2) |
| Bromhexine (intravenous) | 536 (23.9) |
| N-acetylcysteine (nebulized) | 89 (4.0) |
| Fudosteine (oral) | 67 (3.0) |
| Bromhexine (oral) | 54 (2.4) |

Patients were administered the above as a stand-alone agent or in combination.

**Table S5**. Distribution of the number of mucoactive agents administered

|  | |
| --- | --- |
| Different number of mucoactive agents | n (%) |
| 1 | 1646 (73.3) |
| 2 | 461 (20.5) |
| 3 | 128 (5.7) |
| 4 | 10 (0.5) |
| 5 | 1 (0.0) |
